# The ROCKs on which tumour cells thrive

**DOI:** 10.7554/eLife.14511

**Published:** 2016-03-07

**Authors:** Simon Wilkinson, Margaret C Frame

**Affiliations:** CRUK Edinburgh Centre, MRC Institute of Genetics and Molecular Medicine, University of Edinburgh, Edinburgh, United Kingdoms.wilkinson@ed.ac.uk; CRUK Edinburgh Centre, MRC Institute of Genetics and Molecular Medicine, University of Edinburgh, Edinburgh, United Kingdom

**Keywords:** rho kinase, ROCK, cellular senescence, cell proliferation, actomyosin contractility, actin cytoskeleton, Mouse

## Abstract

A new study reveals that the ROCK proteins play key roles in the formation of tumours in mice.

**Related research article** Kümper S, Mardakheh FK, McCarthy A, Yeo M, Stamp GW, Paul A, Worboys J, Sadok A, Jørgensen C, Guichard S, Marshall CJ. 2016. Rho-associated kinase (ROCK) function is essential for cell cycle progression, senescence and tumorigenesis. *eLife* **5**:e12203. doi: 10.7554/eLife.12203

**Image** ROCK proteins help actin filaments in cells to contract (credit: Georgios Kanellos)

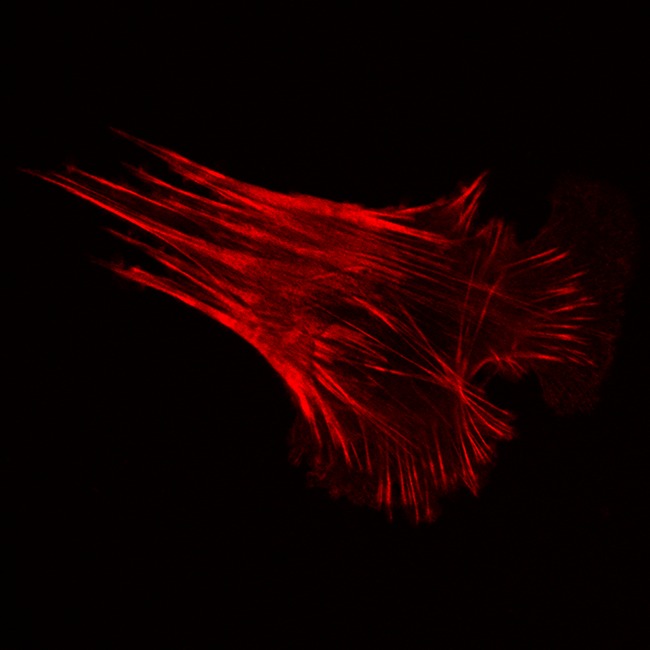

A family of signalling proteins called the Rho GTPases are known to have a profound influence on the formation of tumours in humans and other animals. Once activated, Rho GTPases bind to and activate a network of other proteins in cells, including the enzymes ROCK1 and ROCK2 (Rho-associated, coiled-coil-containing protein kinases; Riento and Ridley, 2003). Now, in eLife, Sandra Kümper of the Institute of Cancer Research (ICR) and colleagues – including the late Christopher Marshall – report that ROCK activity is essential for tumours to form in mice (Kümper et al., 2016).

Cells contain networks of filaments made of a protein called F-actin and the ROCK proteins target other proteins that regulate the accumulation of F-actin. They also regulate the activity of a motor protein called myosin II, which generates the forces that drive the contraction of F-actin via a process called “actomyosin contractility”. This process is critical for, among other things, muscle contraction and the migration of cells around the body.

In part due to two decades of insightful work by Christopher Marshall and various colleagues, it is now accepted that the ROCKs (Sahai and Marshall, 2003) and related kinases (such as the MRCKs; Wilkinson et al., 2005) have key roles in the migration of tumour cells (Sadok et al., 2015). Changes in actomyosin contractility are involved in these migrations, which are responsible for the spread of cancer around the body. Moreover, recent studies show that ROCK1/2 also have other roles in cell division and in promoting tumour growth (Samuel et al., 2011; Kumar et al., 2012).

However, we do not fully understand how the ROCK proteins regulate actomyosin contractility and cancer cell division, or if these phenomena are linked. These are particularly pertinent questions given that clinical trials are underway to test whether some drugs that inhibit the ROCK proteins could be used to treat cancer.

Kümper et al. – who are based at the ICR and Cancer Research UK institutes in London and Manchester – set out to address these questions using mice that carried “conditional” alleles of the genes that encode ROCK1 and/or ROCK2. This made it possible for the team to selectively delete these genes without affecting animal development. First, they isolated fibroblast cells from mouse embryos and created sublines of cells that were unable to produce one or both ROCK proteins. The loss of either ROCK protein alone had no effect on the cells, but the loss of both suppressed actomyosin contractility and stopped the cells from dividing (Figure 1).

**Figure 1.**
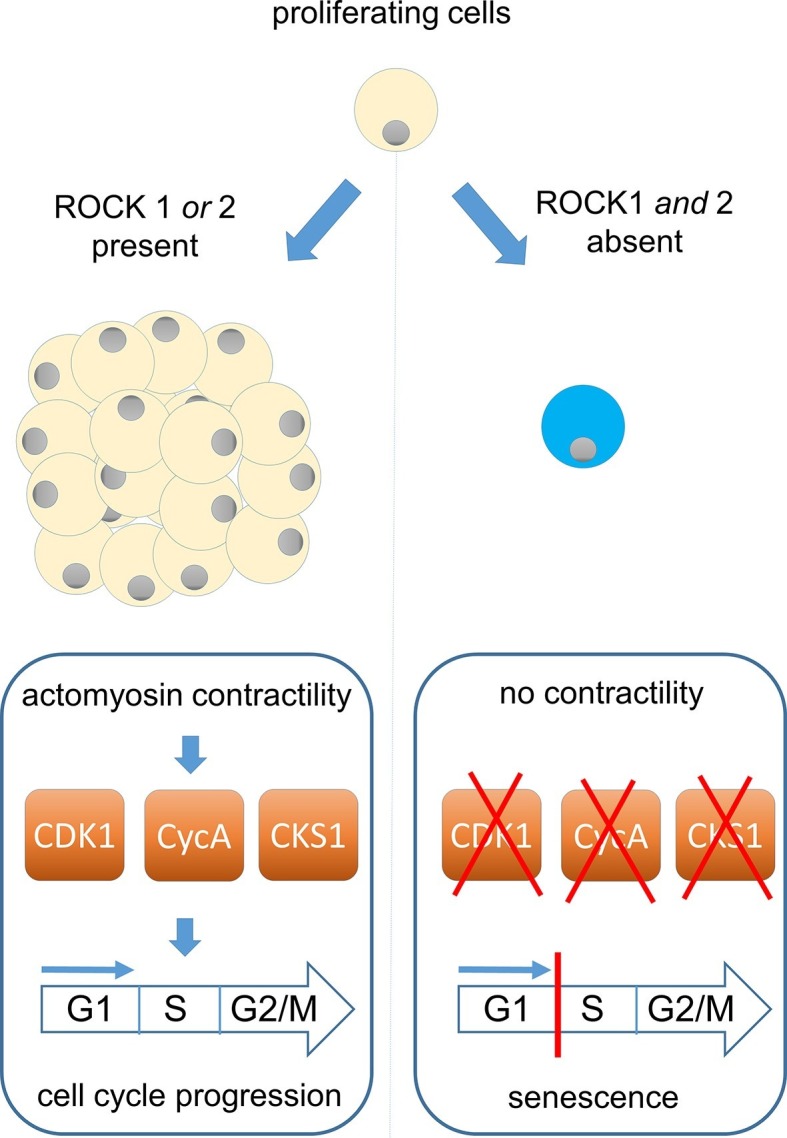
ROCK proteins and the cell cycle. ROCK1 and ROCK2 help cells to divide by enabling a process called actomyosin contractility (left). This process also promotes the production of three proteins (CDK1, CyclinA and CKS1) that have an important role in causing cells to increase in number (proliferate). These proteins drive the cell cycle that underpins the proliferation of both normal and cancerous cells: DNA replication occurs in stage S of the cycle, with cell division taking place in stage G2/M. Kümper et al. found that the loss of both *ROCK* genes, or the use of drugs that inhibit ROCK activity, can permanently stop mouse cells from dividing (right). This phenomenon, which is known as cellular senescence, is probably caused by the loss of actomyosin contractility, which leads to lower levels of CDK1, CyclinA and CKS1.

A drug called Blebbistatin, which inhibits the activity of myosin II, also suppressed cell division. Strikingly, the loss of division in both the cells from the conditional mice and those treated with Blebbistatin was linked to the activation of a new form of cellular senescence, the process by which cells permanently stop dividing (Figure 1). Proteomic analyses revealed that this senescence was hallmarked by specific reductions in the levels of particular cell-cycle proteins. Unusually, this form of senescence did not rely on a key protein that is usually involved in such responses, the tumour suppressor p53.

The above data are exciting because they imply that sustained activity of either ROCK1 or ROCK2 – and the resulting actomyosin contractility – is sufficient to allow cultured cells to continue to divide past the point where they would usually enter senescence. Senescence is also emerging as a major barrier to the formation and growth of tumours in the body: so, does this mean that ROCK proteins are also redundant in tumour formation?

To address this question, Kümper et al. generated cancerous forms of fibroblast cells from the embryos of ROCK conditional mice. The ability of these cells to form tumours was tested by xenografting the cells onto “nude” mice that lack the immune responses that usually protect mice from tumours. The presence of either ROCK1 or ROCK2 was sufficient to support tumour growth, but tumours did not form if both were missing.

Cross-breeding the conditional mice to other mice that were genetically engineered to develop lung cancer showed that the presence of either ROCK1 or ROCK2 alone was sufficient to allow tumour cells to divide. However, the loss of a single ROCK protein alone did have some subtle (and as yet unexplained) effects on cell division. Importantly, Kümper et al. never found any tumours that developed without one or other ROCK protein being expressed, which is consistent with the findings of the xenografted cell line experiments.

An outstanding question is whether the cell senescence phenotype observed in the cell lines would also be seen in the animal. Kümper et al. found that treating mice with a drug called AT13148 – which inhibits both ROCK1 and ROCK2 (Sadok et al., 2015) – could induce the senescence of skin cancer cells that had been grafted into mice. This suggests that it is possible to induce senescence in tumour cells in an animal by targeting the activity of the ROCK proteins. This finding is also consistent with emerging evidence that ROCK inhibitors may have anti-tumour effects (Kumar et al., 2012; Smit et al., 2014). This study extends our understanding of small GTPase signalling pathways and how they contribute to the formation of tumours.
